# Why do faculty resist change?

**DOI:** 10.15694/mep.2021.000089.1

**Published:** 2021-04-08

**Authors:** Clark Dana, Burke Soffe, Jeff Shipley, Frank Licari, Ross Larsen, Kenneth Plummer, Seth Bybee, Jamie Jensen

**Affiliations:** 1 Roseman University of Health Sciences; 2 Brigham Young University

**Keywords:** Dental education, faculty change, professional development, resistance

## Abstract

This article was migrated. The article was marked as recommended.

**Background:** Much of what an educator needs to know to be successful is invisible to lay observers, leading to the assumption that teaching requires little formal study.

**Aims:** This study is based on an 8- month faculty development workshop on student-centered teaching. Faculty members who made no noticeable changes in their teaching practices were compared to faculty who made noticeable and significant changes.

**Method:** Using a qualitative narrative approach based on a structured interview we aimed to categorize the features of changers and resisters.

**Results:** Faculty resisters did not see any need for changes in the way we teach, did not believe student-centered teaching to be more effective, could not appropriately define student-centered teaching, were motivated by extrinsic factors, and felt unvalued. Conversely, faculty changers were excited for changes and saw the need for change and for student-centered teaching, were intrinsically motivated, and felt valued as faculty members.

**Conclusion:** We hypothesize that a main reason for resistance is the status quo bias. Implications for faculty development are discussed.

## Introduction

Efforts to improve teaching are not unique to dental education. A systematic review examining teaching effectiveness in health professions education determined that faculty members in general lack formal training in educational methodology and pedagogy. The review emphasized how faculty development is needed ( Steinert *et al.*, 2006).

Furthermore, cognitive psychologists aptly summarize a major critique of higher education: “It would be difficult to design an educational model that is more at odds with current research on human cognition than the one that is used at most colleges and universities” ( Halpern and Hakel, 2003, p. 4). Yet many faculty members seem unaware of this research or resist giving it serious attention.

As Derek Bok, president *emeritus* of Harvard, has put it, “No faculty ever forced its leaders out for failing to act vigorously enough to improve the prevailing methods of education. On the contrary, faculties are more likely to resist any determined effort to examine their work and question familiar ways of teaching and learning” ( Bok, 2009, p. 334).

Much of what an educator needs to know to be successful is difficult to quantify, leading to the assumption that teaching requires little formal study. If a faculty member has acquired the knowledge of a certain discipline (e.g., dentistry), we assume they are qualified to teach. However, it has been shown that faculty teach in the way they themselves were taught, “using instinct, trial, and personal experience” ( McAndrew, Motwaly and Kamens, 2013, p. 716). With half of new faculty members coming into dental education from private practice, and 18-21% join after graduating from an advanced education program, dental schools must find ways to develop teaching expertise.

Complicating the lack of formal education training for dental educators is the vigorous and frequent reminders that effective pedagogy has evolved, and what they have done in the past is outdated and needs to improve. This article proposes an explanation for individual resistance (or acceptance of) nontraditional educational pedagogies. The focus of the article is how faculty perceive administrative calls for improved teaching and how this process influences individual resistance of new or changing pedagogies. Procedures for decreasing individual resistance to (and, hence, increasing acceptance and use of) active learning methods are suggested.

## Methods

This study is based on an 8-month faculty development workshop project in which faculty members committed themselves to learning about and implementing student-centered teaching in their existing courses. The workshops were scheduled between January 2017 and June 2017 and were endorsed by the Office of Academic Affairs, who requested that all full-time faculty, adjunct faculty, and hygienists attend all sessions of the series. Participants were observed before and after the workshop by trained observers using the Classroom Observation Protocol for Undergraduate STEM (COPUS) ( Smith *et al.*, 2013). Those who made noticeable changes were considered ‘acceptors’ and those who made no change were considered ‘resisters’. (Individual COPUS data is not presented in this manuscript, but is included in a different study, currently unpublished.) While research from this workshop indicated that detailed and directed pedagogical training could cause a change in instructor teaching practice from teacher-centered to more student-centered methods in some, we sought further understanding about those who did not change.

For the purpose of gathering information we used a qualitative narrative approach based on a structured interview with participants of the faculty development workshop. Twenty faculty members participated in all of the workshops. We chose from among the many faculty who made significant changes to be our faculty acceptors group. Only four faculty members made no changes at all and made up our faculty resisters group. Two of these four agreed to participate in our interview process as faculty resisters.

The interviews took place 4 months after the completion of the faculty development workshop series. The privacy of the informants was achieved by receiving their written permission to record the interviews and by strict use of pseudonyms in all written transcriptions. All faculty members were asked the same questions before researchers completed thematic analysis (See Table 1):

• How did you teach before the faculty development workshops?
• How has your teaching changed since the faculty development workshops?
• What is your motivation to be an educator?
• What are your concerns with student-centered teaching?
• What evidence would convince you that student-centered teaching is good?
• What evidence would convince you student-centered teaching is bad?
• What was your opinion going into the faculty development workshops?
• What would you change about the faculty development workshops?
• What resources should be provided to dental educators?
• What incentive structure should be in place for dental educators to transition their classroom to be more student-centered?
• How would you help someone else who is resistant to student-centered teaching?

**Table 1.** Interview questions asked prior to intervention

QUESTION
How did you teach before the faculty development workshops?
How has your teaching changed since the faculty development workshops?
What is your motivation to be an educator?
What are your concerns with student-centered teaching?
What evidence would convince you that student-centered teaching is good?
What evidence would convince you student-centered teaching is bad?
What was your opinion going into the faculty development workshops?
What would you change about the faculty development workshops?
What resources should be provided to dental educators?
What incentive structure should be in place for dental educators to transition there classroom to be more student-centered?
How would you help someone else who is resistant to student-centered teaching?
Other

All interviews were recorded and transcribed according to guidelines established by the Institutional Review Board. Using transcription notes, a thematic analysis was completed on each of the interviews. The procedure for this analysis required researchers to create themes that emerged from the data rather than establish groupings according to an existing theory. The data were read and then reread to categorize teacher views. Similarities and differences across and within participants were examined.

In order to avoid subjectivity in the initial selection of categories and to assure trustworthiness, these themes were identified by each of the four researchers separately, who then compared notes and agreed upon a list of preliminary categories of responses. The final themes were determined at a second and third phase of discussion. The primary author trained the researchers in the method before they began thematic analysis.

## Results/Analysis

Generally speaking, faculty resisters did not see any need for changes in the way we teach and did not believe student-centered teaching to be more effective, while faculty acceptors were excited for changes and saw the need for change and for student-centered teaching. Furthermore, those resisting change did not know the meaning of student-centered teaching (even though this was the focus of the workshop). It was also noted that faculty resisters were motivated by extrinsic factors (salary and recognition), but did not feel valued. On the other hand, faculty acceptors were intrinsically motivated, and felt valued as a faculty member. See Table 2.

**Table 2.** Themes identified through thematic analysis

FACULTY RESISTERS	FACULTY ACCEPTERS
Don’t see a need for change	Excited, and see a need for change
Don’t know what SCT is	Want to know what SCT is
Don’t think SCT is better	Recognize SCT is better
Extrinsic Motivation	Intrinsic Motivation
Don’t feel valued	Feel valued

Within each of the themes noted above were comments illustrating the frustration felt by some faculty that what they are doing is no longer relevant. Regardless of the clinical expertise they bring to their students, the vigorous and frequent reminders that effective pedagogy has evolved, and what they have done in the past is outdated and needs to improve created communication barriers and affected buy-in for what they see as unnecessary mandates from administration.

### A Need for Change?

Faculty acceptors were excited, grateful, and appreciative of the pedagogical training. These faculty sensed that students were hungry for reform, and that traditional models of education were not effective. Furthermore, they even suggested there should be accountability for those faculty unwilling to change their teaching methods.

In contrast, faculty resisters saw no reason to make any changes. Comments from these faculty suggest a weariness and frustration with change efforts, indicating that they felt like the system did not need fixing. These faculty also mentioned their teaching experience and the awards they had received as evidence of their effectiveness. One faculty member indicated change mandates stemming from dental educators with additional degrees were “dangerous.” Example quotes from interviews are shown in Table 3.

**Table 3.** Example quotes regarding the need for change

FACULTY RESISTERS - Don’t see a need for change	FACULTY ACCEPTERS - Excited, and see a need for change
“I rebel against [being asked to change]. I’ll be quite honest with you. But as I say that, I don’t think there isn’t room for improvement. Um, but my opinion is that if, I hate to say this, if it’s not broken, let’s not fix it.”	“I was excited. Like when the Dean said we are going to transform education, I was excited. Yeah! Let’s do this!”
“One of the problems in dental education is that a lot of things are being changed just for the sake of change. They start changing things just for the sake of changing them, and they throw things away that are really good.”	“I am grateful to this school, the dean, and this workshop! It’s made a difference for me and I really appreciate it, even though it’s been a lot of work.”
“I’m going to say something that’s going to throw you for a spin. I have had a lot of experience, and personally I think that dentists get higher degrees in education, and start applying those things in dental education, and I think it can get a little bit dangerous.”	“I was in private practice for 25 years before I came here. And, it’s like, if you are not meeting the outcomes you are out of the game. You are out of business. You are done. The education model is not like that. It’s like you can sleep and lumber along under the radar for your whole career. I think there needs to be some consequence.”
“My opinion is that if it’s not broken, let’s not fix it.”	“It seems students are hungry for something different.”
“I was offered a job and I made an immediate impact, which I expected because I’ve had ten years of teaching experience.”	“There is an extreme need for reform.”
“that is what is so puzzling to me, is why, if we were doing so well (residency acceptance, board scores), do we have to shift gears totally in what we’re doing?	“My motivation was, I had always felt, personally I had always felt like something is wrong with education. We’re not getting the most out of it, or it’s not as effective as it could be.”
“I was applauded at my previous institution.”	“There should be some accountability for not changing.”
“[At my previous institution] I got positive regard from fellow faculty and administrators all the time. Makes me wonder, “why the heck did I leave there?”	
“[At a previous institution] I was given an award five years in a row by the students for excellence in teaching. Since coming here I have also received those awards. What that tells me is that my method works.”	

### Understanding Student-Centered Teaching

After the faculty workshop, those incorporating a more student-centered approach were quick to admit their need to learn more about effective pedagogy. Those maintaining traditional methods seemed to not yet understand the principles of student-centered teaching, either admitting that that was the case or claiming to have already adopted the methods (which was contrary to data collected via classroom observation protocols). Example quotes from interviews are shown in Table 4.

**Table 4.** Example quotes regarding understanding of student-centered teaching

FACULTY RESISTERS - Don’t know what SCT is	FACULTY ACCEPTERS - Want to know what SCT is
“Student-centered vs. teacher-centered. That makes no sense to me.”	“I have so much to learn. I want to be better.”
“I’m already using all the methods being taught in this workshop.”	“I want to be a good teacher, but I need help.”
“I’m under the impression that people think [the way students learn] has changed, but I don’t know if I buy that.”	“When I first came here to teach, I would sit through other lectures and realize, this is painful!” When I started doing my own research into education I realized there was a much better way. I just didn’t know how to start.”
	“I have a lot to learn, but I do know that we learn by doing!” You don’t ride a bike by talking about it. You get on it, you fall down, you get up and you do it again.”
	“My only concern is that I have so much to learn! I need to get better at how I implement [active learning] and do it.

### Confidence in Student-Centered Teaching

During the faculty development workshop series, evidence was presented to highlight the research behind student-centered teaching. Despite the presentations, not all were confident in the research. Faculty acceptors referenced the positive experiences they had with students, and the positive comments made by students. They mentioned the data and were confident the new method was effective. On the other hand, faculty resisters were not confident in the approach and worried that the new teaching styles would not effectively prepare students for their board exams. Example quotes from interviews are shown in Table 5.

**Table 5.** Example quotes regarding confidence in student-centered teaching

FACULTY RESISTERS - Not convinced SCT is better	FACULTY ACCEPTERS - Recognize SCT is better
“It seems we are being asked to throw away everything we’re doing and let’s be entertainers to these students.”	“When I taught using the new techniques [taught in the workshop], the students loved it! They asked me, ‘why didn’t we do it this way sooner?’”
“things are trying to get so accelerated and so streamlined, so to speak, that we’re leaving out some real fundamental things.”	“The research is clear that lecture doesn’t work.”
“[Active learning] has some positive attributes. There are some that go a little further than I’m willing to go. I’ve always done it my own way.”	“The way I was taught was PowerPoint and ‘read the book.’ And then you go to class and are just expected to regurgitate what you just read. I know I personally learn through discovery, but that is not how we teach.”
“I’m just worried about the board results, and I’m worried about the students.”	“There is so much data out there! I mean, going back to the early 1900’s that lecture is not an effective way to deliver material that is going to be retained long term.”
“We may be facing a problem with the board, with a failure rate. I have had a 100% pass rate with the board, but now I don’t know what is going to happen. I’m predicting a higher failure rate on the boards.”	“I am so excited to improve and change. The learning model here is focused on active learning. That is what drew me to this school.”
	“I knew [lecture] wasn’t the best.”
	“After changing my class, students expressed appreciation. They knew that there was effort placed in the teaching method vs. just putting out a PowerPoint lecture and talking about it.”
	“With this class I had so much more feedback than before. Students were like, ‘wow, this has been awesome.’”
	“It was a lot of work to change my class, and sometimes I was like, ‘ahhhhhhhhh!’ But once you get the feedback from students, OK, it was worth it.”
	“[My supervisor] and the Dean have been very forthcoming in their praise for the changes I am making, and that is rewarding. But the reason I am changing is because I feel like it’s just a better way that learning happens. That is why I got into education in the first place.”

### Motivation

The faculty who adopted student-centered methods made comments suggesting they were more intrinsically motivated. For example, they wanted to be better teachers and mentioned doing what was best for the students. Those rejecting these changes were more motivated by extrinsic factors such as salary and recognition. Example quotes from interviews are shown in Table 6.

**Table 6.** Example quotes regarding motivation for change

FACULTY RESISTERS - Extrinsic Motivation	FACULTY ACCEPTERS - Intrinsic Motivation
“I think money would be a good incentivize”	“I love working here at [this school]. I love wanting to be better, I wanted to be an asset to the University, so I had to step out of my comfort zone.”
“As an incentive, I think that monetary, and a rank advancement would be a good incentive.”	“I was initially resistant to change because I was scared, but I realized how much I care about the students. That is what motivates me to improve.”
	“If I were to discover that there is something that I am doing that could be improved, then I would do it. I don’t know that getting an award from the Dean, something that I could put on my door or my wall or whatever, is going to motivate me.”
	“So my main motivation would probably be... I just feel [the students] learn better.”
	“My biggest motivator is internal drive for excellence, and then the second biggest is I need to provide for my family.”

### Feeling Valued

Whether or not the faculty felt valued at their institution was also a theme that emerged from the interviews. Those who changed their teaching practices felt valued while those who did not change their teaching did not. Furthermore, these faculty members mentioned a negative culture. Example quotes from interviews are shown in Table 7.

**Table 7.** Example quotes regarding the value they feel from their institution

FACULTY RESISTERS - Don’t feel valued	FACULTY ACCEPTERS - Feel valued
“There has been no recognition here”	“I feel valued here at [this school], and that makes me want to improve.”
Regarding feedback: “There has been nothing positive.”	“Some people may be motivated by monetary compensation, but I think a bigger motivation for most people would just be recognition that you are valued. I have felt that here.”
“It’s been negative [at this institution] for sure”	
“Well, I’ll tell you, one thing that would really help is if an administrator would come up to me and say, ‘hey I heard you had a good [...] course this year. Way to go! I’m glad you’re here.’”	
“[Positive praise] has never happened here. Never! Not once.”	

## Discussion

After the 8-month faculty development workshop, our research indicated that detailed and directed pedagogical training could cause a change in instructor teaching practice from teacher-centered to more student-centered methods for many faculty participants. Specifically, twenty-one of the twenty-five participants made noticeable changes in their teaching practices. In this project, we chose to utilize a narrative analysis to better understand the perspective of those rejecting change efforts and to compare them with the majority of participants who made changes.

The emerging themes from our analysis led us to the theoretical explanations offered by the status quo bias ( Samuelson and Zeckhauser, 1988), which is a preference for leaving things as they are. Changes in pedagogy hope to improve student learning, but some faculty only see the loss of what they have always done. The faculty members we interviewed who did not change were quick to mention their teaching experience and teaching awards as evidence of the effective status quo. That they were also motivated extrinsically by recognition allows us to gain insight into what they might lose if they were asked to change what they had always done. They might lose the recognition that had brought them to where there were now.

Why is the status quo bias so powerful? Samuelson and Zeckhauser (1988) theorized that it is often an effort to resolve cognitive dissonance, especially in terms of one’s own worth as a decision maker. Asking a faculty member to change how they teach is perceived as an attack on how they have always taught. Past choices are rationalized, even when new evidence suggests improved methodology. The status quo bias limits our ability to change, and the evidence indicates that it applies at least as powerfully to college professors as to any other segment of the population ( Tagg, 2012).

Many of our faculty, however, accepted student-centered teaching, so it is important to ask, “why did only some of the faculty fall prey to the status quo bias?” As reported above, we found that faculty with more experience were less likely to change their teaching practices after pedagogical training and to resist student-centered teaching. This finding is consistent with the research reviewing the literature on the development of pedagogical knowledge for educators ( Prosser and Trigwell, 2001). This data leads us to hypothesize that more experienced faculty have a stronger status quo bias when introduced to pedagogical change.

How can we create a career path for faculty who are afraid of losing their present successes? Case studies detailing change efforts within dental education have indicated some success in implementing large scale changes throughout a curriculum ( Nadershahi *et al*., 2013). A review of these case studies in light of the emergent themes from our interviews would direct us to (a) discover solutions that involve faculty in discovering the perceived need for change, (b) help faculty understand the outcomes of student-centered pedagogies, and (c) properly motivate and value faculty in their ongoing efforts. The following recommendations are provided in view of our findings:

• Frequently engage faculty in the process of change. Dedicate the time required to adequately discuss the reasons and benefits of reform.
• Clearly define what it means to reform and come to a consensus. Discuss what it means to reach these benchmarks and how success will be gauged. Appoint an appropriate entity to gather data and report back to faculty.
• Recognize faculty for outstanding contributions to student-centered teaching, and value these contributions equally with research and service when evaluating academic promotion.
• Secure sufficient resources to support faculty as they reform, including academic promotion incentives and faculty development costs.

## Conclusion

A narrative analysis allows us to focus on an individual perspective and is relevant when that story might be validated by a greater audience. With the ongoing discussion to change dental education and the subsequent findings of faculty resistance to these changes, this approach was seen as an appropriate investigation into pertinent barriers to change for faculty resisters. Understanding the restrictive pull of the status quo bias can give insight to overcome it as an obstacle for change.

## Take Home Messages

• Faculty development does not always lead to noticeable changes.
• Faculty who resist change are likely unaware of the need for change, unaware of student-centered approaches, motivated by external factors, and perhaps undervalued by their peers.
• Faculty who embrace change are likely aware of and excited about change, interested in learning new student-centered techniques, motivated intrinsically to be a better teacher, and perhaps valued by their peers.
• A lack of change is likely motivated by a status quo bias.

## Notes On Contributors

**Dr. Clark Dana** is the Director of Student Assessment, Roseman University of Health Sciences, College of Dental Medicine.

**Dr. Burke Soffe** is the Director of Curriculum, Roseman University of Health Sciences, College of Dental Medicine.

**Jeff Shipley** is a student at Brigham Young University and hopes to attend medical school.

**Dr. Frank Licari** is the Dean at Roseman University of Health Sciences, College of Dental Medicine.

**Dr. Ross Larsen** is an Assistant Professor in the Instructional Psychology and Technology Department at Brigham Young University.

**Dr. Kenneth Plummer** is a Consultant at the Center for Teaching and Learning at Brigham Young University.

**Dr. Seth Bybee** is an Associate Professor in the Biology Department at Brigham Young University.

**Dr. Jamie Jensen** is an Associate Professor in the Biology Department at Brigham Young University.

## Declarations

The author has declared that there are no conflicts of interest.

## Ethics Statement

All interviews were recorded and transcribed according to guidelines established by the Roseman University of Health Sciences (RUHS) Institutional Review Board (IRB), Ref. PN# 16-SJ-DM-1103. Additionally, the ethics and consent process and the study protocol was reviewed and approved by the RUHS IRB, Ref. PN# 16-SJ-DM-1103.

## External Funding

This article has not had any External Funding
