# Supplementary material for: Why do faculty resist change?
Source: MedEdPublish (2016). 2021 Apr 8;10:89. [Version 1] doi: 10.15694/mep.2021.000089.1 (PMC10939649; doi:10.15694/mep.2021.000089.1)
Supplement: Peer review history

# Reviewer report: Reviewer response for version 1

**DOI:** 10.21956/mep.18882.r26951

**Published:** 2021-04-28

**Authors:** Mohamed Al-Eraky

**Affiliations:** 1 University of Dammam

This review has been migrated. The reviewer awarded 3 stars out of 5 Thank you. This is an interesting study on exploring resistance of faculty members to implement change in their practice. First, I concur with all comments of my esteemed colleague, Dr Subha Ramani, particularly in her notion of defining and measuring ‘success’ and the application of Kotter’s model for change, as in this article in Academic Medicine: https://www.researchgate.net/publication/5874037_Faculty_Development_as_an_Instrument_of_Change_A_Case_Study_on_Teaching_Professionalism Then, I would like to share a couple of reflective points. Perhaps it’s not about resistance or acceptance. There are different contextual variables that need to be considered to drive change in a particular setting. Many faculty members might be interested, motivated and wish to plan student-centered sessions, but they couldn’t do it properly due to other reasons. Authors may suggest helpful tips to ‘facilitate’ change, using approaches like community of practice, mentoring programs, or something like a student-centeredness support unit Also, we have different tastes in education and not all SPICES might be used with the same degree in different contexts. There’s no such thing as an ideal international recipe for student-centeredness that’s applicable in Asian, Arabian and Western contexts. For instance, PBL needs to be tailored in view of many variables related to faculty abilities and students’ readiness, as indicated in this article. https://drive.google.com/file/d/0ByugEmLvxFlUcjdLVnlSdlcxZUU/view?usp=sharing Finally, change is not an ultimate aim. Sometimes we may need to reflect on what is suitable for our teachers and students. We may not need a drastic reform, but perhaps a slight deviation from the ‘status quo’. Thank you and good luck!

Reviewer Expertise:

NA

No decision status is available

# Reviewer report: Reviewer response for version 1

**DOI:** 10.21956/mep.18882.r26952

**Published:** 2021-04-09

**Authors:** Subha Ramani

**Affiliations:** 1 Harvard Medical School, Brigham and Women's Hospital

This review has been migrated. The reviewer awarded 4 stars out of 5 I really enjoyed reading this paper about faculty development acceptors and rejectors. I agree with most of the statements made by the authors regarding status quo, assumption that a professional degree is adequate to be a teacher and the fact that educational research and advancements frequently escape the notice of health professionals.The methodology is sound and appropriate to answer the study questions, the themes reported will resonate with many faculty development leaders. I concur with the recommendations provided :engage faculty in the process of change, discuss the 'why' of reform, reach consensus, decide how success is defined, institutional recognition and allocation of resources.My comments are:- the numbers are very small to make broad conclusions. Only 2 rejectors participated. I hope the authors will expand their study and include more participants. I feel more reasons and challenges might come to light.- not sure status quo bias alone is the major factor. Not being valued comes up, thus institutions bear a lot of the blame in why teachers resist change- Motivation is definitely an important, self-determination theory could be a good framework for future research- Institutional culture and behavior principles apply very well, the underlying culture needs to change (difficult though it might be)- Finally, application of Kotter's steps for change might be useful.In summary, I am indicating that individuals should not bear the brunt of these findings, institutions have a major responsibility to create a climate and culture conducive to change and recognise and reward teachers.The article is easy to read and follow and will be useful to all health professions educators and faculty development leaders.

Reviewer Expertise:

NA

No decision status is available
